# Training medical students: victim’s perceptions of selectively screening women for intimate partner violence in health care settings

**DOI:** 10.1186/s12909-019-1627-6

**Published:** 2019-06-11

**Authors:** Olufunmilayo I. Fawole, Busola O. Balogun, Adebola A. Adejimi, O. J. Akinsola, Jacqueline M. Van Wyk

**Affiliations:** 1 Department of Epidemiolgy and Medical Statistics, College of Medicine, University of Ibadan, Ibadan, Nigeria; 2 Department of Community Medicine, College of Medicine, University of Ibadan, Ibadan, Nigeria; 3 Department of Community Medicine, College of Health Sciences, Ladoke Akintola University of Technology, Osogbo, Nigeria; 4 Department of Community Medicine and Primary Health Care, College of Medicine, University of Lagos, Lagos, Nigeria; 5 Department of Clinical and Professional Practice, Nelson R. Mandela School of Medicine, University of Kwa-Zulu Natal, Durban, South Africa

**Keywords:** Intimate partner violence, Screening for intimate partner violence, Health care settings and intimate partner violence, And Women’s perceptions on screening at health care facilities, Nigeria, Africa

## Abstract

**Background:**

Routine IPV screening is a controversial topic and there is no evidence to suggest that it improves the health outcomes of women. Consequently, understanding the socio-cultural dimensions, becomes essential to ensure that victims receive appropriate and local support. This study was conducted to gather the perceptions of victims of IPV on the relevance of raising the topic at health care facilities and to determine specific categories of women to target for screening by medical personnel. It also explored how the information gathered could support victims and whether medical students should be trained on issues relating to IPV.

**Methods:**

Thirty-three key informant interviews were conducted among women attending clinics from three teaching hospitals in the Lagos, Oyo and Osun States of South West Nigeria. The hospitals offer antenatal, emergency, primary care and community outreach clinics which are well-attended by women. A six-item questionnaire assessed eligibility for participation in the study and participants were then purposively sampled. Interviews were conducted using a semi-structured guide. Ethical approval and gatekeepers’ permissions were obtained, and each participant signed informed consent. Data was collected between June and November 2017. The data was entered into Excel and analysed deductively to answer each objective.

**Results:**

Most (*n* = 24) participants stated that medical practitioners should ask all women who present to health care facilities, about their experiences of IPV. Physically, medically and socially vulnerable women, including those in relationships with men in risky occupations, were identified as needing special attention and possible follow-up. They supported the use of the information within and outside of the health care facility, depending on the need of the woman. The majority (*n* = 24) indicated a need to train medical students about IPV and 19 participants suggested for the topic to be curriculated. Most victims favoured the inclusion of a multidisciplinary team in teaching medical students about IPV.

**Conclusions:**

Victims of IPV were in support of initiatives to discuss the topic among some groups of female patients in health care settings. They thought it would enhance the quality of care (medical, psychological, legal and social) to victims. They identified an inter-professional team of stakeholders to include when training medical students about IPV.

## Background

In recent years, attention has increasingly focused on Intimate Partner Violence (IPV) due to the high prevalence, adverse health consequences, culture of silence around the social evil and the potential to prevent its recurrence [1]. International studies report that males are the perpetrators of physical violence in 15–71% of reported cases [2]. Intimate partner violence can impact the wellbeing of women and lead to physical, mental and reproductive health problems. Consequently, it limits the participation of women in society, and causes great human suffering. Women as survivors of IPV often seek medical attention at health care facilities and have identified medical doctors as the health care professionals from whom they are most likely to seek help [3, 4].

Screening practices for IPV at primary care level can facilitate the well-being of women in this vulnerable group. There is currently insufficient evidence to support the implementation of screening programmes in health services generally or in specific clinical settings. Additionally, there is also insufficient evidence to confirm the benefits of screening procedures in reducing incidences of IPV and its impact on the quality of life or health outcomes of survivors [1]. The World Health Organization (WHO) does not recommend universal screening for signs of IPV among women that attend health care centers. It does, however, encourage health care providers to initiate discussions with women suspected of IPV-related injuries and conditions. In settings with limited resources, the WHO recommends a selective approach to screening on the basis of clinical and diagnostic considerations [5]. The WHO has accordingly provided guidelines to enable health care practitioners to respond appropriately to women who have experienced violence, by addressing their emotional and physical health, ongoing safety and support needs [5]. In addition, researchers have recommended the use of qualitative studies to explore women’s needs in relation to screening interventions, and the use of cohort studies to measure the risk factors, resilience factors and the lifetime trajectory of partner violence [6]. Additional research is needed particularly in different local settings around the world because strengthening of health systems and appropriate screening can help providers to address violence against women in a more comprehensive manner [7].

Researchers in the United States have found that screening may provide an opportunity for helpful interventions such as counselling and referral for women with recent histories of IPV [8]. A study in the United Kingdom similarly found that most women patients considered screening an acceptable practice (range 35–99%), although they identified some challenges with screening. The reported challenges included a lack of knowledge on how to screen and issues around the time needed for screening [6]. Likewise, a study of indigenous women in Australia, reported that pregnant women were more likely to disclose abuse, when they perceived health personnel demonstrating caring qualities and an interest in their patients. Given this finding the authors emphasised the importance of discussing abuse with pregnant women to encourage their disclosure of IPV to ensure that they receive appropriate help and support [9]. Approximately 80% of all female patients seen in a hospital in Germany approved of routine inquiry into domestic violence as part of the medical history-taking protocol of the Emergency Department [10]. Relatedly, many studies reported positive results from evaluations of IPV screening programmes particularly to identify victims of abuse [6, 8, 9]. However, the extent to which these programmes can prevent future episodes of abuse remain unknown. The substantial variations reported across studies regarding intervention characteristics, study methodologies, and outcome measures also limited researchers’ ability to make conclusive recommendations on the optimal use of screening interventions [11].

Approximately 20 to 40% of Nigerian women experience IPV, including physical and sexual forms of violence, per year [12]. However, only 31% of the women who experienced these forms of IPV sought help [12]. Consequently, the perceptions of Nigerian women on screening for IPV in health facilities were unknown.

There is thus a need for local studies to assess women’s perceptions of screening as a possible intervention especially since routine IPV screening is a controversial topic and there is little evidence to link the practice to improved health outcomes in women.

Given the understanding of the socio-cultural dimensions, including women’s perceptions, it becomes necessary to ensure that victims receive locally appropriate support for their safety and well-being [13–15]. To this end, this study envisioned to provide evidence to address this gap in knowledge among women in South West Nigeria. The study was conducted to identify the perceptions of victims of IPV to sensitise physicians to the relevance of initiating the topic of IPV at health care facilities. In addition, the research sought to identify specific categories of women to be targeted for inquiry. Finally, it explored the suggestions of victims on the possible use of information gathered during screening and their recommendations on whether and how medical students should be trained to address issues relating to IPV.

## Methods

### Study setting and design

The study was conducted in the three public tertiary health facilities located in Lagos, Oyo and Osun States of South West, Nigeria. The tertiary health facilities or teaching hospitals provide specialist training in disciplines which include Community Medicine, Family Medicine, Obstetrics and Gynaecology, Dentistry, Psychiatry and Accidents and Emergency. The hospitals offer antenatal, emergency, primary care and community outreach clinics which are well attended by women and therefore present a possible location where screening can occur.

Intrinsically, screening in health care settings aims to identify victims and offer support appropriate to their needs. Screening includes a range of methods, involving specific inquiry about IPV or general inquiry about IPV as part of screening [3, 16]. Screening can be done by using a validated screening tool or simply by asking one or a range of questions related to IPV. It is envisaged that screening may increase awareness and educate victims and thus prevent further abuse and possibly reduce consequent problems related to IPV.

This explorative, qualitative case study collected qualitative data from women (*N* = 33) who had experienced physical, psychological and/or sexual violence from a current or former intimate partner. A purposive sampling technique was used to identify eligible participants from women who had attended clinics from three teaching hospitals in the aforementioned states of Southwestern Nigeria.

### Study population

Possible respondents were identified in the health care setting through a review of their medical complaints and based on their physical examinations. The eligibility criteria included that the respondent was an adult i.e. 18 years and older; the respondent was female and that she must have had prior experience of abuse by an intimate partner. Their eligibility to participate was confirmed through a questionnaire. The eligibility instrument was based on questions from the Universal Violence Prevention Screening Protocol, which enabled the documentation of each participants’ experience of physical, sexual and psychological violence over different periods prior to the day of screening [17].

### Study instruments

A semi-structured interview guide was developed, informed by literature on instruments used for screening of victims of IPV [3, 9, 16, 18, 19]. The guide collected socio-demographic information including age, marital status, education, occupation, ethnicity, religion, number of children and identification of the perpetrator. The reliability coefficient of the instrument using the Cronbach alpha test was 0.8. A pilot study, to standardise the instrument, was conducted with four women who attended a private health care facility in Ibadan, Oyo State.

In addition, the 13-item interview guide, explored the extent of previous help-seeking behaviour of victims, their perceptions on whether medical practitioners should screen all or selected women for IPV and their recommendations for the use of gathered information. Additionally, it explored victims’ perceptions of other stakeholders who could assist victims of IPV and their opinions regarding the training of medical students on issues relating to IPV.

The qualitative methodology allowed for the exploration of women’s views on this sensitive topic. It facilitated the exploration of their perceptions on the implementation of screening at health care facilities. The interviews, being face-to-face, could be suspended if deemed inappropriate or if the victim showed signs of distress. This method enabled the interviewer to develop a relationship with respondents and thereby allowed for the collection of comprehensive information from participants on screening for IPV [20].

### Data collection

The data was collected between June and November 2017. The data collection was facilitated by three research assistants, who were trained by the primary investigator on securing and ensuring participant anonymity and confidentiality. The questionnaires were administered in English and Yoruba, the local language. The screening tool took about 20 min to administer. The questionnaire was administered by a research assistant in a consulting room in the health care facility. Only two patients declined to be interviewed after having been found to be eligible to participate. They were afraid of the consequences if their partners were to discover their involvement in the study.

Each interview took about 60 min to complete. Data was collected from 33 participants. This number was deemed sufficient when discussions on the issues did not generate any new themes. After discussion and agreement, the researchers (OF and JV) concluded that saturation had been reached [21].

### Data management

The qualitative data collected was stored on a mobile phone. Codes were assigned to ensure the confidentiality of the participants. Interviews were transcribed and stored electronically in a Microsoft Word Document file. The recorded data was transcribed, cleaned and deductively coded i.e. in response to the main objectives of the study. To code the qualitative data, the two authors (OF and JV) independently read through the transcripts and used highlights to assign initial broad codes to the texts. Next, the deductive analysis resulted in discussions of the framework in relation to the questions that had been posed (theoretical considerations) in the structured interviews. The researchers then came to a common understanding or made meaning of expressed views [21], for example, whether victims expressed opinions indicating that they were in favour of screening or not, and or their reasons for or against screening. The process of analysis was facilitated through the use of Excel [22] and ATLAS.ti [23] software packages.

### Ethical considerations

Ethical approval was obtained from the following institutions: The Oyo State Ministry of Health Ethical Review Committee (AD13/479/165), the University of Ibadan/University College Hospital Joint Institutional Review Board (UI/EC/15/23/13) and from the University of Kwa Zulu-Natal Humanities and Social Science Research Ethics Committee (HSS/1447/015D). Gatekeepers’ permissions to access the facilities were obtained for each institution. The purpose of the study was explained to the participants before conducting the interviews, and they were equally informed of their rights to decline further participation at any stage. Participants were also assured of their right to withdraw and that it would not affect the quality or their access to medical care in any way. Those who elected to participate signed an informed consent form. Importantly, participants were reassured about the confidentiality and anonymity of their responses. Responses were not seen by anyone apart from the investigation team. The interviews were kept in a secure compartment in the custody of the investigators. The investigators had no conflict of interest and the results of this study did not influence their work in any way. Data was entered into a password-protected computer which was stored in a locked cabinet.

## Results

### Characteristics of the participants

Table 1 presents the socio-demographic characteristics of the participants. The mean age of the participants was 35.9 ± 8.01 years. Many had secondary (33.3%) and tertiary (54.5%) education. The participants’ occupations included trading (42.4%), professional work (nursing, legal practitioners) (15.2%) and working in the civil service (2.2%). Most participants (63.6%) were of the Christians faith. Just more than half were married (51.5%), while 33.4% were either separated or divorced. Perpetrators of violence were mainly current or former husbands (90.9%).

**Table 1:** Participants characteristics (*N* = 33)

Characteristics	Total n (%)
Mean age ± SD	35.9 ± 8.0
State	
Lagos	12(36.4)
Osun	12(36.4)
Oyo	9(27.2)
Education	
No formal	1 (3.0)
Primary	3 (9.2)
Secondary	11 (33.3)
Tertiary	18 (54.5)
Occupation	
Trading	14 (42.4)
Artisan	5 (15.2)
Civil servant^a^	7 (21.2)
Professionals^b^	5 (15.2)
Students	2 (6.0)
Religion	
Christianity	21 (63.6)
Islam	12 (36.4)
Ethnic group	
Yoruba	31 (93.9)
Igbo	2 (6.1)
Marital status	
Single	3 (9.1)
Cohabiting	1 (3.1)
Married	17 (51.5)
Separated	9 (27.3)
Divorced	2 (6.1)
Widowed	1 (3.0)
Mean life birth ± SD	2.6 ± 1.1

^a^ = civil servant included teaching ^b^ = professionals included nursing and legal practitioner

### Intimate partner violence prevention screening

The eligibility of the participants that were interviewed was assessed. Subsequently it was noted that all participants had experienced either physical, sexual and/or psychological forms of violence. As shown in Table 2, the fear of physical harm was the most common in the lifetime, previous year and previous month with 72.7, 39.4 and 36.4% respectively. The victims reported that the violence had been perpetrated either by a current or former intimate partner. The second most frequent violent acts included being slapped, pushed, grabbed or shoved.

**Table 2:** Intimate partner violence prevention screening (*N* = 33)

Screening items	Lifetime n (%)	Last 12 months n (%)	Last 1-month n (%)
Being afraid that a current or former intimate partner would cause physical harm	24 (72.7)	13 (39.4)	12 (36.4)
Being slapped, pushed, grabbed or shoved	24 (72.7)	13 (39.4)	8 (24.2)
Being choked, kicked, hit or pushed	19 (57.6)	10 (30.3)	6 (18.2)
Being prevented from leaving the house, seeing friends, getting a job, or continuing with education	16 (48.5)	13 (39.4)	4 (12.1)
Being forced or coerced into sex	12 (33.4)	5 (15.2)	2 (6.1)
Being threatened with or actually using a knife or gun	4 (12.1)	3 (9.1)	0 (0.0)

Ten participants reported having sought police and/or legal protection. Three had sought shelter from family/friends and three admitted that they had separated from their partners following a violent episode.

### Should doctors screen for IPV during the consultation?

Twenty-four (73%) participants stated that practitioners should ask all women who present at health care settings, about their experience of IPV. They thought that screening would not only help those women who might be afraid to speak out, but that it may offer solutions to stop the cycle of violence and to enable holistic care to victims of abuse. Some of their statements are quoted below:

*“Yes, so that their treatment can be holistic. Women can easily confide in their doctors” (B_007).*

*“Yes, because some women come to the clinic with complaints which are different from their real problem which is regular battering from their partners. Victims may be placed on wrong medications if the correct diagnosis is not made” (C_012 and C_07).*

*“Yes, so as to offer solutions before the condition gets out of hand” (A_001).*

Quite differently, some respondents did not support the idea that all women should be screened for IPV. They thought that screening should only be done with women who showed specific signs that suggested abuse. Among the reasons offered by those who disagreed with the doctor-initiated inquiry was the concern about possible emotional trauma to the victims and the possibility that questions may trigger further arguments with abusive partners.

*“No, because it is a family affair, asking victims about it can trigger mental health problems in such women” (B_002).*

*“No, because sometimes it could cause problems or women who have arguments with their partners may begin to complain all the time to the doctor in the hospital” (C_009).*

### What categories of women should be screened?

As indicated in Table 2, almost all participants (29) stated that all women who were married or in relationships should be screened for abuse. Moreover, they suggested that screening should also be extended to three categories of vulnerability highlighted below:

- Physically or medically vulnerable women, such as those presenting to the health care facility with injuries, high blood pressure; or those who are mentally unstable.
- Socially vulnerable such as those women with infertility, those who are pregnant or nursing, homemakers, women of reproductive age, separated/divorced women and those with previous experience of IPV, and
- Those in relationships with men in high-risk occupations such as drivers, vendors and hawkers (someone who moves around selling goods usually informally in public spaces and typically advertises them by shouting).

### Use of information gathered during screening for IPV

Responses regarding the use of the information gathered revealed the following major categories. Firstly, the categories revealed for use either within or outside the health facilities, namely:

i) *Use within the health care facility*: It was recorded that the information should be used to guide patient referral, for victim counseling, to form support groups for women, provide mental health care for victims, to give immediate medical care and to prescribe treatment.

ii) *Use outside the health care facility*: Equal in relevance, it was suggested that the information should be used for legal purposes, to support those in need of evidence and in cases of recurrence of IPV, research purposes to raise awareness on abuse, documentation in order to study patterns of IPV, to create media awareness and to guide non-governmental organisations (NGOs) on how to help victims and conduct seminars for women.

The participants suggested that the information, irrespective of the health care facility, be used to inform the:

i.) Treatment and care to victims: Both involve medical treatment, referral, and the establishment of inter-professional teams (counsellors, human-right activists, etc.) to help women in need.

ii) Education/Training: This is concerned with the organisation of seminars or provision of awareness programmes, for other patients; health education programmes for victims, partners, other women and the society at large.

iii) Counseling/ Advice/ Advocacy: This entails providing guidance to victims, survivors partners and married people.

These were stated as follows:

*“The doctor can advise the woman and give her the necessary treatment. But to prevent death the doctors can refer the woman to a place where she would get help”.*

*(C_003).*

*“They should advise and counsel them. They can organise seminars for women too”.*

*(C_008).*

*“The doctors and nurses can also include the topic in health talks for patients who come to the hospital or clinics” (A_006).*

The participants included a range of suggestions on other possible stakeholders to include, when assisting victims after they had been identified at the health care facility. The stakeholders that were most frequently cited included family members, social workers, nurses, teachers, parents, religious leaders, in-laws, lawyers and law enforcement agents (police). Other less frequently mentioned stakeholders were journalists and non-governmental organisations, foreign organisations, government, psychologist/psychiatrists, local chiefs (Baale) and neighbours.

### Training medical students to screen for IPV

Twenty-four participants supported IPV training for medical students. Four participants did not participate, and five did not respond to this question. Those who responded embraced the stance that medical students should “*be trained in listening”* (C_001).

Another participant stated, *“They should give victims time to express themselves; be taught procedures to identify and treat victims and that training would enable students to offer help to victims of IPV in their own neighborhoods*” (C_006).

Those who did not support the training of medical students, indicated that training "*is not needed as it can make matters worse, and that*… “*the medical student can complicate the matter.*” (B_007).

The most common suggestion (19) in support of the training of medical students was for the inclusion of the topic in the school curriculum. Other suggestions included the *“use of lectures by their lecturers or teachers; posting on work-based learning; small group discussions and offering awareness programmes.”* Participants also thought medical students could be guided through a set of interview questions. They suggested partnerships with professionals, including lawyers, journalists and nurses to assist with the training.

*“The training should be inculcated in their curriculum” (B_010).*

*“During their lecture period, their lecturers can teach them. They can interview those women who have such challenges and learn from them” (C_003.)*

*“Law enforcement agents, lawyers, teachers, lecturers, nurses, social workers and NGO’s can help” (B_004).*

*“Non-medical professionals such as social workers can assist with training the medical students” (B_005).*

## Discussion

This paper describes a study that explored the perceptions of victims of IPV about screening for violence by physicians and other health care practitioners and the use of the information gathered during screening in the health care settings. We also report on recommendations by victims on how medical students should be trained to address issues relating to IPV as future professionals. Almost all the participants indicated the importance for screening of vulnerable women citing both victim and professional reasons.

Women who have been victimised may be unaware of behaviours that constitute abuse or they may actively be seeking support to change their partners’ behaviour [5]. Abused women may also find it difficult to share their experiences, disclose the abuse and seek help. Studies indicate that women who were aware of being abused, were often hesitant to disclose such abuse for personal (embarrassment, fear of retaliation, economic dependency) and social (gender power imbalance in society, family privacy, victim-blaming attitudes) reasons [13, 14]. It is thus necessary to consider that a response to their needs may involve assistance from medical and other health care professionals [17].

The concerns of those who did not think that medical personnel should intervene, or screen for IPV should also be considered. It is important that provision be made to manage women who may have an emotional breakdown during the screening/consultation process. Precaution should also be taken to ensure the safety of both the victims and the health care practitioners from possible aggressive partners.

There is much controversy about the benefits and harm of screening for IPV in health care settings. A large evidence-based review reported no evidence of the benefits, costs or potential harm on the use of screening to improve women’s health and well-being. The study report preferred a case-finding approach to assess patients based on their risks for (and/or clinical signs or symptoms of) exposure [6]. However, an updated systematic review in the United States concluded that screening practices can identify abused women as some intervention studies had shown promise. They recommend for the inclusion of universal screening protocols at health care settings, considering that screening for IPV would vary by population and setting [24].

Two large trials, in Canada [25] and in the USA [26] confirmed that health care practitioners should be alert to signs and symptoms associated with IPV. They recommended for practitioners to ask about possible abuse when presented with clinical findings of possible abuse. They recommended for screening to be done sensitively and to respond to the women’s needs and safety concerns. Health care settings are furthermore advised to develop and implement protocols to appropriately refer abused women to local services as per the identified need of the individual [27]. The commonality across studies that report women’s fears of being harmed and evidence of physical abuse on a global scale, highlight the need for action which extends beyond the provision of a health care service provision but also to collaborate to prevent violence against women [28].

The severity of the experiences of IPV among participants in this study varied. Some necessitated police and legal interventions, which resulted in separation from abusive partners. Women exposed to severe physical or psychological IPV were found to be more likely to involve law enforcement such as the police than women who suffer other forms of abuse [29]. The identification and accurate classification of IPV are therefore important especially for women who need to be separated from abusive partners. An accurate classification system could inform future decisions about the care of their children and whether to separate or divorce from abusive partners. Such a system was reportedly helpful to researchers and mental health practitioners to design appropriate interventions for survivors and their families [30].

The typical obvious consequences of physical violence include bruising, abrasions, burns, fractures, head injuries and spinal cord damage [17, 31]. Emergency medical physicians report that the proportion of women with physical injuries who seek emergency medical care, were typically fewer than those who sought help for psychological complaints [32]. Psychological violence, particularly verbal abuse, was also a common complaint of women in our study.

Other studies have identified psychological violence as the most common form of IPV experienced by women [12]. Given the many consequences of psychological violence [33], health care providers should therefore be aware of signs and symptoms of psychological violence when treating women. Complaints of anxiety, depression, sadness, insomnia, irritability, confused thinking, feelings of extreme elation or sadness, dramatic changes in eating or sleeping patterns, fear of intimacy, loss of self-esteem and self- respect and anger should serve as possible indicators of spousal abuse [1]. Also, frequent psychosomatic complaints such as pain in the head, back, breast, abdomen, gastrointestinal disorders and disturbances in menstruation and reproductive health were found to be suggestive of abuse and should be investigated [34, 35].

The identification of vulnerable women is especially important for practitioners working in resource-constrained settings that typically have fewer personnel, time to dedicate to patients and limited referral options. However, before screening is advocated in health care settings, health care personnel need to be competent to respond appropriately to abused women [3]. The WHO recommends that health facilities have the necessary policies and protocols for the management of victims and resources in place to enable them to refer patients to non-governmental organisations and community-based organisations for support prior to screening for IPV. In addition, the health care facility should format clinical guidelines and offer skills training to practitioners. It would be deemed unethical and potentially harmful to commence screening on IPV without having these measures in place at health care settings [5, 7].

Considering the limited resources in the African setting, the selective enquiry would be more appropriate for use in local health care settings [5]. The selective enquiry is recommended for women with physical and mental health problems, women with infertility, pregnant or nursing women, homemakers, separated/divorced women and those with previous experience of IPV. Pregnant women are well recognized as being a vulnerable group to IPV [2]. These extant studies recommended that mothers are asked about IPV at antenatal clinics as the practice has been reportedly helpful to ensure adequate referral and services to stop the cycle of violence among prenatal women in the United States, New Zealand, and Sweden [36]. Studies have also recommended the use of selective enquiry for women with sexually transmitted infections [37], living with HIV and AIDS [38], and those who choose to induce an abortion [39, 40].

A noteworthy suggestion from participants in the current study was for information obtained from victims to be used both within and outside the health care setting. Preventing violence against women requires a multi-sectoral approach, in which the health sector plays a central role. This strategy includes the early identification of abuse, providing victims with the necessary treatment, and referring women for appropriate and informed care [8]. An effective strategy will reduce maternal morbidity and mortality and it will promote family health by preventing child abuse. Recommendations for the management of victims of IPV include advocacy, counseling and support from other groups based at the health care facility.

A systematic review of 12 trials found that interventions such as advocacy and cognitive behavioural therapy, played a major role in reducing both physical and psychological violence associated with IPV [41]. A significant reduction in IPV victimization was reported following a counseling intervention [42]. These forms of interventions were regarded as beneficial to victims, their partners and members of the public. This multi-sectoral approach to ending abuse should involve governmental and non-governmental organisations, research institutes, the media, and educational organisations. All these stakeholders have crucial roles in providing comprehensive care to the victims of abuse [34].

Apart from medical assistance to victims, the women’s responses confirmed the significance of a multidisciplinary approach to stopping violence against women. Recognised inter-disciplinary (medical) groups such as obstetricians and gynaecologists; internal and emergency medicine physicians; psychiatrists, family and community physicians have launched effective interventions and care, to respond to victims of IPV [3]. Other health disciplines include psychologists, social workers, and nurses, while trans-disciplinary groups such as the police and legal practitioners may also be beneficial in helping women who experience IPV [1, 34] Medical students could learn from these groups strategies, and contribute through mentoring and partnerships.

Most of the victims agreed that medical students should receive training on issues relating to IPV. They suggested that training include the use of an inter-professional approach as reported in previous studies with medical students [43] and stakeholders [44]. Studies in high-income countries found that the training of medical students on gender-based violence can improve the identification of abused women who use the health care facilities [16], however from this study it is unclear what education strategies will be most helpful to victims. It is therefore important for further studies to evaluate existing IPV education programmes and identify the most effective education strategies to promote improved health care and health outcomes for abused women and their families [45]. Confidentiality of victims’ information should be emphasised during training programmes to allay the concerns of women who cited confidentiality as the reason for excluding medical students from training. A previous study had recommended for training be offered only to mature, senior medical students who were thought to be better able to handle sensitive information [44].

Our findings underpin the need for the health sector to take IPV against women more seriously than thus far and to play a greater role in responding to IPV against women. Multiple entry points within the health sector exist, where women may seek health care particularly in sexual and reproductive health services, such as antenatal care, post-abortion care, family planning, mental health and emergency services. These services provide good opportunities to educate women about IPV. One crucial aspect of training for health care practitioners is to identify opportunities to provide support and to link women to other services that they may need [28]. The WHO guidelines on IPV emphasise the urgent need to integrate IPV victim care in upskilling in-service training, and to include the topic in undergraduate curricula for health care professionals. This health sector response needs to be part of a multi-sectoral response [7], as recently endorsed in the Agreed Conclusions of the 57th session of the Commission on the Status of Women [45].

The strength of this study lies in the views gathered from the participants who are the victims of IPV. Not only were the women from different locations and attending different clinics in the training hospitals, but they had experienced different types of abuse over varying periods of time. The participants’ diversity in background and experiences of violence provided better insight into their perspectives across settings, and thus increased our confidence in the results.

The study was unique in that it considered victims’ (patients) perceptions as an important component in the training of the health care practitioners and therefore aimed to obtain their insights on issues such as the training of medical students to prepare them for their future role as medical doctors. In addition, the originality of the work lies in the fact that, to our knowledge, this is one of the first studies that provides qualitative data on this topic in the African context. However, our study has some limitations. The victims were selected from health care facilities that are linked to training institutions and they were therefore perhaps more accepting of screening for IPV and in the training of medical students, than if they were selected from centres in the community. The study participants may also be more familiar with the procedures and challenges of big health care facilities; therefore we also interviewed women attending primary health care facilities and outreach clinics attached to these big hospitals, to include a variety of women with diverse backgrounds and experiences. Despite this, the participants were not representative of the general population of Nigerian women, as most were well-educated and therefore may be more informed about IPV and the health care setting.

From the foregoing, it is obvious that the findings of this study improve our understanding of the target population for selective screening at health care facilities. Moreover, the most important contribution from this study relates to how the information obtained can be used to effectively intervene on behalf of victims of IPV and in the training medical students and in-service health care professionals. Subsequently, it is believed that these recommendations will guide curricular interventions to improve women’s health. This study did not explore the perceptions of male patients and it would be beneficial to explore the perceptions of these victims to screening practices and to include their opinions on the training of staff and medical students in health care settings.

A medical curriculum that improves student’s knowledge and skills on gender-based violence is urgently required. By addressing issues on the identification of victims, their treatment and care, the required support services and including the medico-legal aspects of IPV in the curriculum, medical students will be empowered to intervene on behalf of victims in their future role as medical doctors and to foster trans-disciplinary collaboration. Future research should develop practical and feasible training curricula for African medical students. This curriculum could be developed through a consortium of experts consisting of doctors, social workers, nurses, etc., who, in collaboration with experts from other relevant disciplines and institutions, should develop a protocol for the management of victims of abuse in health care settings. Gender-based violence experts should lead the implementation of training and periodical re-training, in collaboration with non-governmental, governmental and legal organisations, for all health care providers working in critical areas of the health care facility. The development of a register of victims (data collection for active monitoring and quality control) is recommended and a network of assistance for victims after they leave the health care facility.

## Conclusion

Victims of IPV who participated in this study supported the notion that health care practitioners in health care settings in Nigeria should selectively screen women, particularly female patients with physical and mental health problems and social vulnerabilities, for signs of abuse. IPV is addressable in the health care setting, not only via screening but also through provider-based counseling and referral to appropriate legal or social services. Selective screening of female patients within the health care setting may enhance the quality of care provided to women. Most participants thought that medical students should be taught about IPV and they recommended that a multidisciplinary team of experts train medical students accordingly. Health professionals, however, must first acknowledge IPV as a possible cause of injuries and other health complaints in female patients and be prepared to work with professionals from other sectors to address this issue.

## Data Availability

The datasets used and/or analysed during the current study are available from the corresponding author on reasonable request.

## Abbreviations

AIDS: Acquired Immune Deficiency Virus
HIV: Human Immunodeficiency Virus
IPV: Intimate Partner Violence
NGO: Non-governmental Organisation
SD: Standard Deviation
WHO: World Health Organisation
